# Supplementary material for: An interaction graph approach to gain new insights into mechanisms that modulate cerebrovascular tone
Source: Commun Biol. 2024 Apr 3;7:404. doi: 10.1038/s42003-024-06064-1 (PMC10991376; doi:10.1038/s42003-024-06064-1)
Supplement: Supplementary file 2 — Supplementary Information [file 42003_2024_6064_MOESM2_ESM.pdf]

Supplementary Material
An Interaction Graph Approach To Gain New Insights Into Mechanisms That Modulate Cerebrovascular Tone.
Communications Biology

Sergio Dempsey[1,*], **Finbar Argus**[1] **Gonzalo Daniel Maso Talou**[1], **Soroush Safaei**[1]
[1]*Auckland Bioengineering Institute, University of Auckland, Level 6/70 Symonds Street, Grafton, Auckland 1010, New Zealand*
Correspondence*:
Sergio Dempsey
sdem348@aucklanduni.ac.nz

Refer to the IGs regularly to track paths. The acronym table used in the main manuscript is reproduced in the end of this supplementary for keyword / mechanism search efficiency. Several citations link to extensive reviews on topics; interested readers are encouraged to read those sources as well to gain a better understanding, second perspective, and identify supporting experimental results.

SUPPLEMENTARY NOTE 1: MECHANISMS OF IG1

Neurotransmitters

NVC begins with the release of neurotransmitters (NTs) from neurons. NTs we cover include glutamate (Glu), gamma-aminobutyric acid (GABA), acetylcholine (ACh), noradrenaline (NA), adenosine triphosphate (ATP), neuropeptide Y (NPY), and adenosine. Each NT has its own family of receptors, which are membrane-bound proteins with a location for agonist binding shown in Table S1. There are two types of receptor proteins, ionotropic and metabotropic. Ionotropic means that the receptor protein is an ion channel and allows for the exchange of polarising or depolarising ions directly. Metabotropic receptors are receptor proteins that affect the metabolism of other enzymes.

Table S1. NTRs and their respective receptors.

NT	Receptor	Ref.
Glu	ionotropic α-Amino-3-hydroxy-5-methyl-4-isoxazolepropionic acid receptor (AMPAr), ionotropic N-methyl-D-aspartate receptor (NMDAr), metabotropic glutamate receptors (mGlur)	1,2,3,4
GABA	ionotropic GABA-A receptor (GABA-Ar), metabotropic GABA-B receptor (GABA-Br)	2,4,5
ATP	ionotropic P2X and metabotropic P2Y	6,2,4
Adenosine	metabotropic adenosine receptors (mAr)	6,2
NPY	metabotropic NPY receptors (NPYr)	6,5
ACh	ionotropic nicotinic acetylcholine receptor (nAChr), metabotropic muscarinic acetylcholine receptor (mAChr)	6,2,3,4
NA	metabotropic α and β adrenergic receptors ($\alpha_{\#}$Ar and βAr respectively)	7

Receptors

Mechanisms related to cerebrovascular tone (CVT) of receptor proteins in response to agonist stimulation are described in Table S2. Noticeably, the mechanisms described end with activation of: inward rectifying potassium (K^{1+}) channels (K_{ir}), the enzymes phospholipase C (PLC) and enzyme adenylyl cyclase (ACyc), or depolarisation / hyperpolarisation. The enzyme mechanisms are described next, while the depolarisation and hyperpolarisation descriptions are reserved for section* .

Table S2. General Function of each NT membrane receptor.

Receptor	Function
NMDAr	Membrane channel protein opens with glycine or D-serine, making the cytosol permeable to Ca^{2+} (depolarisation)[8,9]
AMPAr	Membrane channel protein slightly permeable to K^{1+}, Ca^{2+}, and fully permeable to Na^{1+}, (net depolarisation)[10]
mGlur	Activates PLC, deactivates ACyc[11]
GABA-Ar	Membrane channel protein permeable to Chloride (Cl^{1-}) (hyperpolarisation)[12]
GABA-Br	Deactivates VOCCs (hyperpolarisation)[12]
P2X	Membrane channel protein permeable to Ca^{2+} (depolarisation)[13]
P2Y	Activates PLC and deactivates ACyc[14,15,13]
mAr	Activates and deactivates ACyc depending on type[13]
nAChr	Membrane channel protein permeable to Ca^{2+} (depolarisation)[16]
mAChr	M(1,3,5) activates PLC (depolarisation), M(2,4) activates $K_{ir}3$ (hyperpolarisation)[17,18]
NPYYr	Deactivates ACyc[19]
$\alpha_{\#}$Ar	α_1 activates PLC, α_2 deactivates ACyc[20], [21],
βAr	Activates ACyc[7],

K_{ir} Channel Activation

The family of K_{ir} channels allow passage of K^{1+}, especially when bound to phosphatidylinositol 4,5-bisphosphate (PIP2)[22]. Certain K_{ir} channels also require activation by membrane-bound G proteins, for example, $K_{ir}3$ which is a strong K^{1+} channel present largely throughout the neurovascular unit[22]. The activation of K_{ir} channels hyperpolarises the cell[22,4].

Phospholipase C Activation

PLC hydrolyses PIP2, splitting to inositol triphosphate (IP_3) and diacylglycerol (DAG), causing multiple mechanisms of depolarisation[23]. First, the removal of PIP2 from a K_{ir} channel will cause depolarisation via K_{ir} closure[17,22]. The produced IP_3 acts on the endoplasmic reticulum (ER) and sarcoplasmic reticulum (SR), releasing Ca^{2+} and causing depolarisation[24]. This Ca^{2+} can bind to ryanodine receptors on the SR or ER if present, which releases more Ca^{2+} and causes further depolarisation. This phenomenon is called Ca^{2+} induced Ca^{2+} release (CICR)[24]. Depolarisation by Ca^{2+} also opens voltage-operated Ca^{2+} channels (VOCC) on the cellular membrane, allowing the entry of extracellular Ca^{2+} causing further depolarisation[25]. The other biproduct of IP3 breakdown, DAG, can activate protein kinase C (PKC)[26]. PKC can inhibit large K^{1+} channels (BK) and affect other ion channels, leading to net depolarisation[27].

Adenylyl Cyclase Activation

The role of ACyc is to convert ATP to cyclic adenosine monophosphate (cAMP). cAMP as a secondary messenger has numerous downstream pathways. The predominant pathway is the activation of protein kinase A (PKA), which affects the mechanics of vascular smooth muscle (VSM) (discussed in more detail in section*). The other major roles of cAMP are to open nucleotide-gated ion channels and enhance NT release[28]. Regarding this stage of mechanisms involving cellular membrane polarity, ACyc activation creates cAMP that depolarises cells.

Calcium Activated Pathways in Neurons and Astrocytes

In response to available NTs and cellular membrane receptors, net depolarisation in neurons and astrocytes will result in an increase in cytosolic Ca^{2+} or a decrease (from hyperpolarisation). On depolarisation, neurons and astrocytes release their own NTs back into the synaptic cleft[29,30,4]. This can affect the original response of signaling neurons, increasing the complexity of possible NTs that act on a cell at any one time. This interaction highlights that multiple cells dictate net-released NTs and vasomodulating products, reaching the VSM.

The remainder of this subsection* will focus on the internal cell response to depolarisation for both neurons and astrocytes. The cumulative impact from all sources will then be described in the VSM.

Arachidonic Acid Pathways

Ca^{2+} stimulates the enzymes phospholipase-A2 (PLA2) and -D2 (PLD2) that produce arachidonic acid (AA). In neurons, the dominant enzyme is PLA2 and in asotrocytes, the dominant enzyme is PLD2, although both are present in both cells[1,2]. AA is first broken down into prostaglandin H_2 (PGH_2) by cyclooxygenase (COX) and peroxidase[31]. In neurons, the dominant enzyme is COX2 and in astrocytes, the dominant enzyme is COX1[32,33]. PGH_2 can then be broken down into numerous prostaglandin metabolites by respective prostaglandin synthases (PGSs) (e.g., predominantly PGE_2 in astrocytes and PGI_2 in endothelial cells) or thromboxane (TX) by thromboxane synthase (TXS).

Epoxyeicosatrienoic acid (EET) is produced from AA by cytochrome P450 epoxygenase (CP450E)[34]. 20-Hydroxyeicosatetraenoic acid (20-HETE) is produced with AA by cytochrome P450 hydroxylase (CP450H)[35]. CP450E is found in both neurons and astrocytes[36], while CP450H is found mainly in VSM[35].

NO pathway

Cytosolic Ca^{2+} can bind to calmodulin (CaM). Fully bound, CaM is an agonist for neuronal nitric oxide synthase (nNOS). nNOS, with L-Arginine, oxygen (O_2), and nicotinamide adenine dinucleotide phosphate (NADPH), produces L-Citrulline, NADP+, water (H_2O), and nitric oxide (NO)[37]. As the name suggests, nNOS is produced in neurons, predominantly GABAergic interneurons[38].

Potassium pathways

In response to Ca^{2+}, depolarisation, concentration of cAMP, PGE2, and EET agonists, astrocyte BK channels open[39]. These channels are permeable to K^{1+} which is released into the perivascular space (PVS) as most BK channels are located on astrocyte endfeet[39]. General neuron activity also releases K^{1+} into the extracellular space, which can diffuse to the PVS[25]. An increase in extracellular K^{1+} can increase the affinity for K^{1+} flux into other cell types, causing hyperpolarisation and the closing of VOCCs.

VSM response

Through mechanisms of diffusion, membrane channel transport, NTs, and stretch (discussed in section*), vasomodulating metabolites and ions are transported to the VSM, which will alter CVT[40]. The mechanical description of the changes in VSM tone is described by[41], which is based on the sliding filament theory of actin and myosin. This theory describes contraction as based on the amount of connected actin and myosin (AM) cross-bridges, which may be phosphorylated (AMp). An AM bridge is produced after an AMp bridge has completed its power stroke and contraction complex. The control of AM phosphorylation is myosin light chain kinase (MLCK). This enzyme is activated by Ca^{2+} bound CaM and is therefore regulated by Ca^{2+}. The mechanism of relaxation is to dephosphorylate AMp bridges, which is controlled by myosin light chain phosphatase (MLCP), or to inhibit MLCK. Through changes in Ca^{2+}, or these enzymes, modulation of VSM cell tone is possible.

Relaxation

The main forms of relaxation are the production of cyclic nucleotides. Several NTs and vasomodulating metabolites control production by activating or inhibiting ACyc and guanylyl cyclase to produce cAMP and cyclic guanine monophosphate (cGMP), respectively. Refer to Table S2 which describes which NTs will activate ACyc. Regarding the metabolites produced by neurons and astrocytes; PGE2/PGI2 acts on its dominant metabotropic receptor EP4 which activates ACyc[2], and diffused NO activates guanylyl cyclase[25]. cAMP and cGMP activate PKA and protein kinase G (PKG)[42]. PKA inhibits MLCK causing relaxation[43,42], while PKG primarily activates MLCP[42]. cGMP and cAMP can also activate each others main protein kinase with 10x larger amount needed for activation[42]. Cyclic nucleotides can also inhibit selective phosphodiesterases that break down other cyclic nucleotides[44,45,46]. This crosstalk and the prolonged longevity of nucleotides lead to increased relaxation.

When transported to the VSM, EETs activate BK channels that hyperpolarise the cell, close VOCCs, and cause relaxation[47]. cAMP, cGMP, PKA, and PKG can also activate K^{1+} channels (voltage dependant, K_{ir}, BK, ATP sensitive, and more) leading to depolarisation[27,39,42]. Lastly, K^{1+} from the PVS can be taken up by VSM K_{ir} channels, causing hyperpolarisation, closing VOCCs and decreasing Ca^{2+}.

Contraction

ATP, NA, NPY, ACh all have receptors on VSM cells[6]. Referring to Table S2, ATP opening P2X increases Ca^{2+} directly, ATP activates P2Y which stimulates PLC causing IP3 Ca^{2+} release, CICR, and VOCC Ca^{2+} increases. NA and NPY deactivate ACyc, decreasing cAMP and leaving MLCK uninhibited. NA can also activate the PLC path[7], and NPY may also enhance IP3r Ca^{2+} release[5]. ACh activates predominant $m_{2/3}$ receptor proteins causing depolarisation[21]. TX acts on TX receptors (TP) on VSM to activate PLC paths, and deactivate cyclic nucleotide enzymes leading to constriction[48,49]. Lastly, AA in VSM can be broken down into 20-HETE. 20-HETE mainly deactivates BK channels leading to depolarisation, although other depolarising pathways are possible[50].

Other Vasomodulating Mechanisms

While the above discussion concludes the NVC pathways from neuronal stimulation, feedforward NVC to VSM is not the only way to locally modulate vessel tone. We now describe other mechanisms related to healthy cerebrovascular tone function.

Endothelial Activity

Endothelial cells are unique in that they also receive signals from the lumen, in addition to signaling from parenchymal cells. Common lumen agonists are ATP, ACh, Angiotensins and mechanical activation (strain and wall shear stress discussed in section*). ATP is released by red blood cells under increased shear stress, which results from increased blood pressure[51]. This ATP opens P2X, and activates P2Y, increasing Ca^{2+} and causing depolarisation. Unlike astrocytes and neurons, free IP3 and Ca^{2+} in the endothelium can be transported directly to the VSM through gap junctions, causing contraction[25]. ACh delivered in blood plasma responds to mAchr's on the endothelium[52]. ACh can also be released by the endothelium as a vasodilating factor into the lumen from increased stress[53]. mAChrs on endothelial cells mainly act to increase Ca^{2+} by activating PLC[21] although they can hyperpolarise by opening K_{ir} channels as well. Some mAChr channels may stimulate phosphotidylinositol-3-kinase (PI3K)[54] discussed in section* . Angiotensins can act on angiotensin receptor (ATr) proteins (commonly AT_1r and AT_2r). AT_1r causes contraction by activating PLC and transporting IP3 and Ca^{2+} to the VSM[55]. AT_2r plays a counter role by opening BK channels and stimulating PI3K to activate the NO pathway[56,57]. The dominant role depends on the expression of the receptor and protein and is likely spatially dependent.

Endothelial cells also have receptors for NTs that may not originate from the lumen. ATP, ACh, and other signals from perivascular nerves or transport of parenchymal metabolites can also act on endothelial cells[21]. Adenosine receptors that activate ACyc are present in endothelial cells[58] that activate ACyc. βAr receptors (as opposed to predominantly αAr in VSM) are also present, which activate ACyc[7]. Both increase cAMP production and activate the NO pathway.

Lastly, increases in Ca^{2+} causes a similar cascade of enzymatic activity found in neurons and astrocytes. Endothelial cells express endothelial NOS (eNOS) that produces NO which diffuses to the VSM[37]. Endothelial cells also produce AA and subsequent vasodilatory prostaglandins, EET, and vasoconstricting TX; all are available for transport to the VSM[59].

Stretch and Stress Activation

Stretch can modulate CVT by opening stretch-activated cation channels (SACCs), activating PLC, and stimulating NO production. SACCs are present in endothelial cells[25,60], astrocytes[61], and VSM[60,62]. In response to increased stretch (pressure), SACCs open, allowing Ca^{2+} and other positively charged ions into the cell, causing depolarisation. This can also lead to CICR and the opening of VOCCs leading to increased contraction[63]. Two essential SACCs within the VSM are TRPM4 and TRPC6[62,64]. Recent work has identified pathways that involve stretch-sensitive activation of G-proteins through a mechanosensitive G-protein-coupled receptor (GPCR), which then activates PLC and increases production of IP3 and DAG[60]. TRPC6 is hypothesised to be stimulated by DAG and pressure. TRPM4 is hypothesised to increase local Ca2+ in the subsarcolemmal space due to influx through TRPM4, and IP3-induced Ca^{2+} release.

In endothelial cells and astrocytes, TRPV4 ion channels are expected[25,61]. However, in contrast to SACCs in VSM, SACCs such as TRPV4 in endothelial cells have been shown to attenuate the myogenic response of VSM by activating BK_{Ca} channels in the VSM via gap junctions[60]. Recent evidence suggests that these channels may also not be directly stretch-sensitive[65] and therefore are likely activated by an unknown stretch-sensitive pathway[60]. Lastly, important membrane enzymes can be activated by wall shear stress in endothelial cells. Shear stress-stimulated PI3K activates PKA, which in turn activates eNOS, causing dilation[66]. PKA is also activated by shear stress[37].

SUPPLEMENTARY NOTE 2: MECHANISMS OF IG2

Neuronal metabolic effects on NVC are not often considered when discussing NVC and several studies suggest that there is little or no effect[67]. However, these discussions are based on studies that involve oxygen (O_2) or glucose, not the biproduct of oxygen metabolism, carbon dioxide (CO_2). Approximately 20% of the body's energy is consumed by the brain, with neurons accounting for 80%[68]. As neurons consume these large amounts of O_2, they produce CO_2 molecules in a 1 to 1 ratio. Intraluminal CO_2 is a known vasodilator and neural inhibitor[69], so it is interesting that this influence is rarely discussed in relation to CVT from the neuron side. Given recent mechanism literature, we believe that there is enough supporting evidence to hypothesise neuronal CO_2 modulation of CVT.

Transport

Regarding standard CO_2 transport to the lumen, CO_2 passively diffuses from the neuron to the extracellular space through CO_2 channel transporters, where it is converted to carbonic acid by carbonic anhydrase (CA). Carbonic acid then readily dissociates into bicarbonate and a free hydrogen atom[70]. Extracellular bicarbonate is then taken into astrocytes primarily through electrogenic sodium-bicarbonate cotransporters (NBCe1) junctions into astrocytes[71]. Nonconverted extracellular CO_2 can also diffuse through connexin hemichannels into astrocytes[72]. Within astrocytes, cytosolic bicarbonate is converted back into CO_2 and water by astrocyte CA[71]. Astrocytic CO_2 then diffuses through aquaporin-4 channels on astrocyte endfeet[69] into the PVS and from there, CO_2 is assumed to diffuse through the endothelium to the lumen.

Mechanisms of Hypercapnia Induced Vasodilation

It is well observed that an increase in CO_2 causes an increase in cerebral blood flow[45], and it was recently reported that CO_2 impacts NVC independent of acidosis, suggesting CO_2 dependent mechanisms[69]. This is important, as the general consensus has been that CO_2 mainly drives changes in pH (increased carbonic acid) that alter the membrane channel and enzyme activity[45,73] without accounting for the acidosis independent CO_2 contribution, which will be addressed in the following.

For pH-dependent mechanisms in all cells, acidosis increases the activity of ATP-dependent K^{1+} channels[45] and decreases the activity of VOCCs[74]. In neurons, there are also acid-sensing ion channels (ASICs) that cause depolarisation[73]. This activity is particularly interesting, as K^{1+} channel activity and VOCC inhibition decrease cell activity, but ASICs cause an active vasodilating process by releasing NO[73]. A positive feedback loop is also formed as NO increases the activity of ASICs[75]. In fact, this active effect is so prominent that ASIC knockout reduced the magnitude of cerebral blood flow response to hypercapnia by 70% when inhaling 5% and 10% CO_2 (in rats)[73]. Interestingly, the same magnitude reduction in hypercapnia-induced vasodilation could be repeated by inhibiting NOS, suggesting that ASIC predominantly activates nNOS. We suspect that since the expression of ASICs is lower in pyramidal neurons[73], there is a balance between K^{1+} channel and VOCC inhibition versus ASIC activation. In interneurons, increased expression of ASIC activates abundant interneuron nNOS pathways[73].

Large systematic reviews on the effect of CO_2 agree with the above that NOS is clearly a driver of hypercapnia-induced vasodilation, but it has also been shown that COX inhibition can provide a nearly identical decrease in the CVT response to hypercapnia and only the combination of NOS and COX inhibition can effectively stop the CO_2 response[69]. An interesting discussion provided in[76] proposes a unifying theory for the COX-NOS dependence through the generation and crosstalk of cyclic nucleotides. NO in the VSM stimulates the production of cGMP as expected, which would then mainly inhibit phosphodiesterase

to prevent the breakdown of cAMP, or activate PKA itself, as opposed to independently activating PKG. Examination of IG1 shows that cAMP is dependent on COX biproducts (through PGE) and unifies both dependencies (discussed in section*s and). In support of the COX-NOS dependence, combined PKA and PKG knockout greatly inhibited the hypercapnic response to CO_2 in rats (reduction from $\approx$70% dilation to $<$5%)[77]. Capillary dilation has also been reported to depend on PGE synthesis, which depends on COX, to activate EP4 receptors that activate cAMP production[33,78]. In Mishra et al.[33], inhibition of NOS did not affect the magnitude of normocapnic neuron-stimulated dilation. Potentially, NOS is only recruited at the capillary level under hypercapnic conditions for vasodilation, or spatial differences between works alter the strength of the mechanisms.

Internal changes in pH can cause internal Ca^{2+} release in endothelial cells[79]. This Ca^{2+} can stimulate the production of eNOS and COX dependent pathways, potentially identifying the COX dependence, but we argue that this is implausible for two reasons. First, changes in internal pH require substantial changes in extracellular pH beyond what is possible without extreme acidosis[80,81]. Within typical ranges of 5% and 10% for common CO_2 study, no changes in internal pH are expected. The second reason is the overwhelming evidence that endothelial disruption does not impact CVT in response to hypercapnia in vivo[82], nor does the endothelium increase NO production in response to hypercapnia, which would also be a consequence of increased Ca^{2+} to drive COX paths[83,84]. However, there is some controversy here, as hypercapnia has been found to cause NO production in cultured endothelial cells[85] and induce vasodilation in excised cerebral arteries[86,87,88]. This is likely caused by differences in experimental conditions; in vivo results suggest that the endothelial influence in minimal. See the review of Hoiland et al.[45] for further reading.

To explain the dependence of COX on hypercapnia, we now turn to CO_2 and begin with the discussion of CO_2 sensitive connexins, which are nonspecific ion channels[89] that are also permeable to ATP (allowing transport and release)[90,91]. The presence of CO_2 sensitive connexins has been identified in adult dopaminergic and GABAergic neurons in the ventral tegmental area, but it is generally not present in other locations of the brain[92,91]. However, these connexins are abundant in astrocytes[93,94]. Astrocytes also have an appreciable concentration of free ATP in the cytoplasm capable of diffusing through these connexins[90,91]. In response to the CO_2-opened connexins, ATP is released into the intercellular space to interact with P2X and P2Y receptors on nearby neurons and form a positive feedback loop on the astrocyte itself. P2X and P2Y will allow the influx of Ca^{2+} and activate PLC pathways resulting in IP3 production, CICR, AA production and finally, COX-based activity and metabolites released to VSM. Supporting the dependence on astrocytes are observations that astrocytic release of ATP is critical for NVC in response to CO_2[95]. Furthermore, damaged astrocytes were shown to attenuate the hypercapnic response by 50%[96].

Some final remarks on the mechanisms; P2Y and P2X receptors are not abundant in interneurons[97], so we hypothesise that pyramidal neurons would primarily be activated by $CO_2 \rightarrow$ ATP dependent mechanisms, while interneurons would be activated primarily by pH-dependent ASIC$\rightarrow$NO mechanisms. Bicarbonate can also activate ACyc within neurons, endothelial cells, and astrocytes, showing that other, Ca^{2+} independent relaxation pathways are also still possible[98]. The release of ATP from astrocytes can also cause depolarisation by activating the ATP-sensitive K^{1+} channels mentioned above.

Mechanisms of Hypercapnia Induced Neural Inhibition

The CO_2 vasodilation has been explained in the previous section*; however, inhibition of neurons and NVC at varying degrees of hypercapnia, independent of pH[69] has not. To provide several possible causes, it is possible that internal pH, which was not measured in[69], caused inhibition[99]. Intracellular CA is

present in all neurons and explains the conversion of CO_2 to bicarbonate and free hydrogen, lowering the pH[100,101]. Another option; bicarbonate is also known to enhance the activity of GABA-Ar receptors[70]. In response to CO_2-ATP release, excited pyramidal neurons and astrocytes release NTs that further excite interneurons, releasing GABA and causing feedback inhibition. This would postulate that the effect of NTs on interneurons would be larger than that of ASIC depolarisation to release excess GABA, or that other mechanisms of GABA release are activated, not covered here.

SUPPLEMENTARY NOTE 3: MECHANISMS OF IG3

The mechanisms of IG3 are as relevant for CVT as those presented in IG1 and IG2. The separation here is to show the benefit of a pre-existing IG and how it can be leveraged to understand literature that does not fully explain mechanisms, and to show how these graphs can be updated as new information is released. In particular, we show that each compound promotes or inhibits existing pathways already covered in IG1. This fills in the blanks where the respective literature did not cover a connected path to VSM tone, or consider the competing or parallel mechanisms that are present on IG1.

Bradykinin is an available lumen delivered vasodilating metabolite that acts on endothelial cells through bradykinin receptor proteins that stimulate PI3K needed for the NO path[102,103,104]. Perivascular nerves release specialised compounds for nerve-mediated CVT modulation. Calcitonin gene-related peptide (CGRP) is an exemplar perivascular nerve metabolite that acts on CGRP receptors activating ACyc-dependent vasodilation[105,21]. Perivascular nerves can also release vasoactive intestinal peptide (VIP)[21]. VIP acts on receptors $VPAC_1$ and $VPAC_2$ which may have differentiated function[106]. In general, VPACs can activate ACyc, PLD2, and possibly PI3K, all leading to potent vasodilation[106].

Other gases beyond NO and CO_2 can modulate CVT. Hydrogen sulphide (H_2S) can alter the open probabilities of K^{1+} channels, help maintain cGMP in the cytosol, and promote the activity of eNOS and PI3K[107]. Carbon monoxide (CO) is a close relative of CO_2 but interacts through different mechanisms leading to vasodilation. Produced by heme-oxygenase (HO) in endothelial cells and astrocytes, CO increases the open probabilities of VOCCs and potassium channels, can promote the release of locally stored endothelial NO, and increase the activity of NOS[108].

There are also sex-dependent factors that contribute to CVT modulation by the influence of hormones and gene expression[109,110]. Estrogen can activate heme-oxygenase[111] and can promote prostaglandin production by activating COX and PGS[109]. Testosterone also promotes prostaglandin production by activating COX, but shifts downstream production to TX, causing vasoconstriction[112]. Testosterone also opens K^{1+} channels, causing hyperpolarisation and potential relaxation[112]. Both testosterone and estrogen activate eNOS[112] and PI3K, which contribute to NO production[113]. Each has an opposing effect on the expression of AT_2r in endothelial cells with estrogen that promotes the receptor protein and testosterone that downregulates it[110]. Changes in available hormones with age are also expected to be an important contributor to CVT, especially after menopause[114]. Testosterone can also be converted to estrogen by aromatase[115] and subject-specific expression may predispose some females to larger changes in CVT.

SUPPLEMENTARY REFERENCES

[1]Attwell, D. *et al.* Glial and neuronal control of brain blood flow. *Nature* **468**, 232–243, DOI: 10.1038/nature09613 (2010).

[2]Hosford, P. S. & Gourine, A. V. What is the key mediator of the neurovascular coupling response? *Neuroscience & Biobehavioral Reviews* **96**, 174–181, DOI: 10.1016/j.neubiorev.2018.11.011 (2019).

[3]Lecrux, C. & Hamel, E. Neuronal networks and mediators of cortical neurovascular coupling responses in normal and altered brain states. *Philosophical Transactions of the Royal Society B: Biological Sciences* **371**, 20150350, DOI: 10.1098/rstb.2015.0350 (2016).

[4]Bazargani, N. & Attwell, D. Astrocyte calcium signaling: The third wave. *Nature Neuroscience* **19**, 182–189, DOI: 10.1038/nn.4201 (2016).

[5]Uhlirova, H. *et al.* Cell type specificity of neurovascular coupling in cerebral cortex. *eLife* **5**, DOI: 10.7554/eLife.14315.001 (2016).

[6]Sweeney, M. D., Kisler, K., Montagne, A., Toga, A. W. & Zlokovic, B. V. The role of brain vasculature in neurodegenerative disorders. *Nature Neuroscience* **21**, 1318–1331, DOI: 10.1038/s41593-018-0234-x (2018).

[7]Xanthopoulos, A., Daskalopoulou, I., Frountzi, S. & Papadimitriou, E. A Systematic Review on the Role of Adrenergic Receptors in Angiogenesis Regulation in Health and Disease. *International Journal of Translational Medicine* **1**, 353–365, DOI: 10.3390/ijtm1030021 (2021).

[8]Stroebel, D. & Paoletti, P. Architecture and function of NMDA receptors: An evolutionary perspective. *The Journal of Physiology* **599**, 2615–2638, DOI: 10.1113/JP279028 (2021).

[9]Skowrońska, K., Obara-Michlewska, M., Zielińska, M. & Albrecht, J. NMDA Receptors in Astrocytes: In Search for Roles in Neurotransmission and Astrocytic Homeostasis. *International Journal of Molecular Sciences* **20**, 309, DOI: 10.3390/ijms20020309 (2019).

[10]Chen, S. & Gouaux, E. Structure and mechanism of AMPA receptor — auxiliary protein complexes. *Current Opinion in Structural Biology* **54**, 104–111, DOI: 10.1016/j.sbi.2019.01.011 (2019).

[11]Spampinato, S. F., Copani, A., Nicoletti, F., Sortino, M. A. & Caraci, F. Metabotropic Glutamate Receptors in Glial Cells: A New Potential Target for Neuroprotection? *Frontiers in Molecular Neuroscience* **11**, DOI: 10.3389/fnmol.2018.00414 (2018).

[12]Pinard, A., Seddik, R. & Bettler, B. GABAB Receptors: Physiological Functions and Mechanisms of Diversity. *Advances in Pharmacology* **58**, 231–255, DOI: 10.1016/S1054-3589(10)58010-4 (2010).

[13]Burnstock, G. Historical review: ATP as a neurotransmitter. *Trends in Pharmacological Sciences* **27**, 166–176, DOI: 10.1016/j.tips.2006.01.005 (2006).

[14]von Kügelgen, I. & Hoffmann, K. Pharmacology and structure of P2Y receptors. *Neuropharmacology* **104**, 50–61, DOI: 10.1016/j.neuropharm.2015.10.030 (2016).

[15]Lohr, C. Role of P2Y receptors in astrocyte physiology and pathophysiology. *Neuropharmacology* **223**, 109311, DOI: 10.1016/j.neuropharm.2022.109311 (2023).

[16]Shen, J.-x. & Yakel, J. L. Nicotinic acetylcholine receptor-mediated calcium signaling in the nervous system. *Acta Pharmacologica Sinica* **30**, 673–680, DOI: 10.1038/aps.2009.64 (2009).

[17]Brown, D. A. Muscarinic Acetylcholine Receptors (mAChRs) in the Nervous System: Some Functions and Mechanisms. *Journal of Molecular Neuroscience* **41**, 340–346, DOI: 10.1007/s12031-010-9377-2 (2010).

[18]Abrams, P. *et al.* Muscarinic receptors: Their distribution and function in body systems, and the implications for treating overactive bladder. *British Journal of Pharmacology* **148**, 565–578, DOI: 10.1038/sj.bjp.0706780 (2006).

[19]Molosh, A. I. *et al.* NPY Y1 Receptors Differentially Modulate GABAA and NMDA Receptors via Divergent Signal-Transduction Pathways to Reduce Excitability of Amygdala Neurons. *Neuropsychopharmacology* **38**, 1352–1364, DOI: 10.1038/npp.2013.33 (2013).

[20]Strosberg, A. D. Structure, function, and regulation of adrenergic receptors. *Protein Science* **2**, 1198–1209, DOI: 10.1002/pro.5560020802 (1993).

[21]Xavier, F. E. Nitrergic perivascular innervation in health and diseases: Focus on vascular tone regulation. *Acta Physiologica* **230**, e13484, DOI: 10.1111/apha.13484 (2020).

[22]Hibino, H. *et al.* Inwardly Rectifying Potassium Channels: Their Structure, Function, and Physiological Roles. *Physiological Reviews* **90**, 291–366, DOI: 10.1152/physrev.00021.2009 (2010).

[23]Lyon, A. M. & Tesmer, J. J. G. Structural Insights into Phospholipase C- β Function. *Molecular Pharmacology* **84**, 488–500, DOI: 10.1124/mol.113.087403 (2013).

[24]Berridge, M. J. Neuronal Calcium Signaling. *Neuron* **21**, 13–26, DOI: 10.1016/S0896-6273(00)80510-3 (1998).

[25]Kenny, A., Plank, M. J. & David, T. The role of astrocytic calcium and TRPV4 channels in neurovascular coupling. *Journal of Computational Neuroscience* **44**, 97–114, DOI: 10.1007/s10827-017-0671-7 (2018).

[26]Schubert, R., Noack, T. & Serebryakov, V. N. Protein kinase C reduces the K_{Ca} current of rat tail artery smooth muscle cells. *American Journal of Physiology-Cell Physiology* **276**, C648–C658, DOI: 10.1152/ajpcell.1999.276.3.C648 (1999).

[27]Jaggar, J. H., Porter, V. A., Lederer, W. J. & Nelson, M. T. Calcium sparks in smooth muscle. *American Journal of Physiology-Cell Physiology* **278**, C235–C256, DOI: 10.1152/ajpcell.2000.278.2.C235 (2000).

[28]van der Horst, J., Greenwood, I. A. & Jepps, T. A. Cyclic AMP-Dependent Regulation of Kv7 Voltage-Gated Potassium Channels. *Frontiers in Physiology* **11**, DOI: 10.3389/fphys.2020.00727 (2020).

[29]Hamilton, N. B. & Attwell, D. Do astrocytes really exocytose neurotransmitters? *Nature Reviews Neuroscience* **11**, 227–238, DOI: 10.1038/nrn2803 (2010).

[30]Sten, Sebastian. *Mathematical Modeling of Neurovascular Coupling* (Linköping University Electronic Press, 2020).

[31]Samuelsson, B. Role of Basic Science in the Development of New Medicines: Examples from the Eicosanoid Field. *Journal of Biological Chemistry* **287**, 10070–10080, DOI: 10.1074/jbc.X112.351437 (2012).

[32]Choi, S. H., Aid, S. & Bosetti, F. The distinct roles of cyclooxygenase-1 and -2 in neuroinflammation: Implications for translational research. *Trends in Pharmacological Sciences* **30**, 174–181, DOI: 10.1016/j.tips.2009.01.002 (2009).

[33]Mishra, A. *et al.* Astrocytes mediate neurovascular signaling to capillary pericytes but not to arterioles. *Nature Neuroscience* **19**, 1619–1627, DOI: 10.1038/nn.4428 (2016).

[34]Spector, A. A. Arachidonic acid cytochrome P450 epoxygenase pathway. *Journal of Lipid Research* **50**, S52–S56, DOI: 10.1194/jlr.R800038-JLR200 (2009).

[35]Imig, J. Epoxyeicosatrienoic Acids and 20-Hydroxyeicosatetraenoic Acid on Endothelial and Vascular Function. *Advances in Pharmacology* **77**, 105–141, DOI: 10.1016/bs.apha.2016.04.003 (2016).

[36]Ferguson, C. S. & Tyndale, R. F. Cytochrome P450 enzymes in the brain: Emerging evidence of biological significance. *Trends in Pharmacological Sciences* **32**, 708–714, DOI: 10.1016/j.tips.2011.08.005 (2011).

[37]Forstermann, U. & Sessa, W. C. Nitric oxide synthases: Regulation and function. *European Heart Journal* **33**, 829–837, DOI: 10.1093/eurheartj/ehr304 (2012).

[38]Tricoire, L. & Vitalis, T. Neuronal nitric oxide synthase expressing neurons: A journey from birth to neuronal circuits. *Frontiers in Neural Circuits* **6**, 1–36, DOI: 10.3389/fncir.2012.00082 (2012).

[39]Girouard, H. *et al.* Astrocytic endfoot Ca $^{2+}$ and BK channels determine both arteriolar dilation and constriction. *Proceedings of the National Academy of Sciences* **107**, 3811–3816, DOI: 10.1073/pnas.0914722107 (2010).

[40]Sweeney, M. D. *et al.* Vascular dysfunction—The disregarded partner of Alzheimer's disease. *Alzheimer's & Dementia* **15**, 158–167, DOI: 10.1016/j.jalz.2018.07.222 (2019).

[41]Hai, C. M. & Murphy, R. A. Cross-bridge phosphorylation and regulation of latch state in smooth muscle. *American Journal of Physiology-Cell Physiology* **254**, C99–C106, DOI: 10.1152/ajpcell.1988.254.1.C99 (1988).

[42]Morgado, M., Cairrão, E., Santos-Silva, A. J. & Verde, I. Cyclic nucleotide-dependent relaxation pathways in vascular smooth muscle. *Cellular and Molecular Life Sciences* **69**, 247–266, DOI: 10.1007/s00018-011-0815-2 (2012).

[43]Horman, S. *et al.* AMP-activated Protein Kinase Phosphorylates and Desensitizes Smooth Muscle Myosin Light Chain Kinase. *Journal of Biological Chemistry* **283**, 18505–18512, DOI: 10.1074/jbc.M802053200 (2008).

[44]Elvebak, R. L., Eisenach, J. H., Joyner, M. J. & Nicholson, W. T. The Function of Vascular Smooth Muscle Phosphodiesterase III is Preserved in Healthy Human Aging. *Clinical and Translational Science* **3**, 239–242, DOI: 10.1111/j.1752-8062.2010.00232.x (2010).

[45]Hoiland, R. L., Fisher, J. A. & Ainslie, P. N. Regulation of the cerebral circulation by arterial carbon dioxide. *Comprehensive Physiology* **9**, 1101–1154, DOI: 10.1002/cphy.c180021 (2019).

[46]Sadek, M. S., Cachorro, E., El-Armouche, A. & Kämmerer, S. Therapeutic Implications for PDE2 and cGMP/cAMP Mediated Crosstalk in Cardiovascular Diseases. *International Journal of Molecular Sciences* **21**, 7462, DOI: 10.3390/ijms21207462 (2020).

[47]Pfister, S. L., Gauthier, K. M. & Campbell, W. B. Vascular Pharmacology of Epoxyeicosatrienoic Acids. *Advances in Pharmacology* **60**, 27–59, DOI: 10.1016/B978-0-12-385061-4.00002-7 (2010).

[48]Nakahata, N. Thromboxane A2: Physiology/pathophysiology, cellular signal transduction and pharmacology. *Pharmacology & Therapeutics* **118**, 18–35, DOI: 10.1016/j.pharmthera.2008.01.001 (2008).

[49]Ozen, G. *et al.* Mechanism of thromboxane receptor-induced vasoconstriction in human saphenous vein. *Prostaglandins & Other Lipid Mediators* **151**, 106476, DOI: 10.1016/j.prostaglandins.2020.106476 (2020).

[50]Hoopes, S. L., Garcia, V., Edin, M. L., Schwartzman, M. L. & Zeldin, D. C. Vascular actions of 20-HETE. *Prostaglandins & Other Lipid Mediators* **120**, 9–16, DOI: 10.1016/j.prostaglandins.2015.03.002 (2015).

[51]Zhang, H., Shen, Z., Hogan, B., Barakat, A. I. & Misbah, C. ATP Release by Red Blood Cells under Flow: Model and Simulations. *Biophysical Journal* **115**, 2218–2229, DOI: 10.1016/j.bpj.2018.09.033 (2018).

[52]Radu, B. M. *et al.* All muscarinic acetylcholine receptors (M1-M5) are expressed in murine brain microvascular endothelium. *Scientific Reports* **7**, DOI: 10.1038/s41598-017-05384-z (2017).

[53]Wilson, C., Lee, M. D. & McCarron, J. G. Acetylcholine released by endothelial cells facilitates flow-mediated dilatation. *The Journal of Physiology* **594**, 7267–7307, DOI: 10.1113/JP272927 (2016).

[54]Dedkova, E. N., Ji, X., Wang, Y. G., Blatter, L. A. & Lipsius, S. L. Signaling Mechanisms That Mediate Nitric Oxide Production Induced by Acetylcholine Exposure and Withdrawal in Cat Atrial Myocytes. *Circulation Research* **93**, 1233–1240, DOI: 10.1161/01.RES.0000106133.92737.27 (2003).

[55]Swiderski, J. *et al.* Role of Angiotensin II in Cardiovascular Diseases: Introducing Bisartans as a Novel Therapy for Coronavirus 2019. *Biomolecules* **13**, 787, DOI: 10.3390/biom13050787 (2023).

[56]Carey, R. M., Wang, Z.-Q. & Siragy, H. M. Role of the Angiotensin Type 2 Receptor in the Regulation of Blood Pressure and Renal Function. *Hypertension* **35**, 155–163, DOI: 10.1161/01.HYP.35.1.155 (2000).

[57]Paz Ocaranza, M. *et al.* Counter-regulatory renin–angiotensin system in cardiovascular disease. *Nature Reviews Cardiology* **17**, 116–129, DOI: 10.1038/s41569-019-0244-8 (2020).

[58]Sancho, M. *et al.* Adenosine signaling activates ATP-sensitive K^+ channels in endothelial cells and pericytes in CNS capillaries. *Science Signaling* **15**, eabl5405, DOI: 10.1126/scisignal.abl5405 (2022).

[59]Félétou, M., Huang, Y. & Vanhoutte, P. M. Endothelium-mediated control of vascular tone: COX-1 and COX-2 products. *British Journal of Pharmacology* **164**, 894–912, DOI: 10.1111/j.1476-5381.2011.01276.x (2011).

[60]Jackson, W. F. Calcium-Dependent Ion Channels and the Regulation of Arteriolar Myogenic Tone. *Frontiers in Physiology* **12**, 770450, DOI: 10.3389/fphys.2021.770450 (2021).

[61]Tureckova, J., Hermanova, Z., Marchetti, V. & Anderova, M. Astrocytic TRPV4 Channels and Their Role in Brain Ischemia. *International Journal of Molecular Sciences* **24**, 7101, DOI: 10.3390/ijms24087101 (2023).

[62]Baek, E. B. & Kim, S. J. Mechanisms of myogenic response: Ca2+-dependent and -independent signaling. *Journal of Smooth Muscle Research* **47**, 55–65, DOI: 10.1540/jsmr.47.55 (2011).

[63]Niggel, J., Sigurdson, W. & Sachs, F. Mechanically Induced Calcium Movements in Astrocytes, Bovine Aortic Endothelial Cells and C6 Glioma Cells. *Journal of Membrane Biology* **174**, 121–134, DOI: 10.1007/s002320001037 (2000).

[64]Schubert, R. & Brayden, J. E. Stretch-activated Cation Channels and the Myogenic Response of Small Arteries. In Kamkin, A. & Kiseleva, I. (eds.) *Mechanosensitivity in Cells and Tissues* (Academia, Moscow, 2005).

[65]Nikolaev, Y. A. *et al.* Mammalian TRP ion channels are insensitive to membrane stretch. *Journal of Cell Science* **132**, DOI: 10.1242/jcs.238360 (2019).

[66]Boo, Y. C. *et al.* Shear Stress Stimulates Phosphorylation of Endothelial Nitric-oxide Synthase at Ser1179 by Akt-independent Mechanisms. *Journal of Biological Chemistry* **277**, 3388–3396, DOI: 10.1074/jbc.M108789200 (2002).

[67]Zhu, W. M., Neuhaus, A., Beard, D. J., Sutherland, B. A. & DeLuca, G. C. Neurovascular coupling mechanisms in health and neurovascular uncoupling in Alzheimer's disease. *Brain* **145**, 2276–2292, DOI: 10.1093/brain/awac174 (2022).

[68]Watts, M. E., Pocock, R. & Claudianos, C. Brain Energy and Oxygen Metabolism: Emerging Role in Normal Function and Disease. *Frontiers in Molecular Neuroscience* **11**, DOI: 10.3389/fnmol.2018.00216 (2018).

[69]Hosford, P. S. *et al.* CO2 signaling mediates neurovascular coupling in the cerebral cortex. *Nature Communications* **13**, 2125, DOI: 10.1038/s41467-022-29622-9 (2022).

[70]Ruusuvuori, E. *et al.* Neuronal carbonic anhydrase VII provides GABAergic excitatory drive to exacerbate febrile seizures. *The EMBO Journal* **32**, 2275–2286, DOI: 10.1038/emboj.2013.160 (2013).

[71]Theparambil, S. M., Naoshin, Z., Thyssen, A. & Deitmer, J. W. Reversed electrogenic sodium bicarbonate cotransporter 1 is the major acid loader during recovery from cytosolic alkalosis in mouse cortical astrocytes. *Journal of Physiology* **593**, 3533–3547, DOI: 10.1113/JP270086 (2015).

[72]Gourine, A. V. & Dale, N. Brain H+/CO2 sensing and control by glial cells. *GLIA* **70**, 1520–1535, DOI: 10.1002/glia.24152 (2022).

[73]Faraci, F. M. *et al.* Acid-Sensing Ion Channels: Novel Mediators of Cerebral Vascular Responses. *Circulation Research* **125**, 907–920, DOI: 10.1161/CIRCRESAHA.119.315024 (2019).

[74]Tombaugh, G. C. & Somjen, G. G. Effects of extracellular pH on voltage-gated Na+, K+ and Ca2+ currents in isolated rat CA1 neurons. *The Journal of Physiology* **493**, 719–732, DOI: 10.1113/jphysiol.1996.sp021417 (1996).

[75]Cadiou, H. *et al.* Modulation of Acid-Sensing Ion Channel Activity by Nitric Oxide. *The Journal of Neuroscience* **27**, 13251–13260, DOI: 10.1523/JNEUROSCI.2135-07.2007 (2007).

[76]Okamoto, H., Hudetz, A. G., Roman, R. J., Bosnjak, Z. J. & Kampine, J. P. Neuronal NOS-derived NO plays permissive role in cerebral blood flow response to hypercapnia. *American Journal of Physiology-Heart and Circulatory Physiology* **272**, H559–H566, DOI: 10.1152/ajpheart.1997.272.1.H559 (1997).

[77]Pelligrino, D. Cyclic nucleotide crosstalk and the regulation of cerebral vasodilation. *Progress in Neurobiology* **56**, 1–18, DOI: 10.1016/S0301-0082(98)00009-4 (1998).

[78]Howarth, C. *et al.* A Critical Role for Astrocytes in Hypercapnic Vasodilation in Brain. *The Journal of Neuroscience* **37**, 2403–2414, DOI: 10.1523/JNEUROSCI.0005-16.2016 (2017).

[79]Ziegelstein, R. C. *et al.* Modulation of calcium homeostasis in cultured rat aortic endothelial cells by intracellular acidification. *American Journal of Physiology-Heart and Circulatory Physiology* **265**, H1424–H1433, DOI: 10.1152/ajpheart.1993.265.4.H1424 (1993).

[80]Asai, M. *et al.* Extracellular acidosis suppresses endothelial function by inhibiting store-operated Ca2+ entry via non-selective cation channels. *Cardiovascular Research* **83**, 97–105, DOI: 10.1093/cvr/cvp105 (2009).

[81]Capellini, V. K., Restini, C. B. A., Bendhack, L. M., Evora, P. R. B. & Celotto, A. C. The Effect of Extracellular pH Changes on Intracellular pH and Nitric Oxide Concentration in Endothelial and Smooth Muscle Cells from Rat Aorta. *PLoS ONE* **8**, e62887, DOI: 10.1371/journal.pone.0062887 (2013).

[82]Wang, Q., Pelligrino, D. A., Koenig, H. M. & Albrecht, R. F. The Role of Endothelium and Nitric Oxide in Rat Pial Arteriolar Dilatory Responses to CO_2 in vivo. *Journal of Cerebral Blood Flow & Metabolism* **14**, 944–951, DOI: 10.1038/jcbfm.1994.126 (1994).

[83]Iadecola, C., Yang, G. & Xu, S. 7-Nitroindazole attenuates vasodilation from cerebellar parallel fiber stimulation but not acetylcholine. *American Journal of Physiology-Regulatory, Integrative and Comparative Physiology* **270**, R914–R919, DOI: 10.1152/ajpregu.1996.270.4.R914 (1996).

[84]Wang, Q., Pelligrino, D. A., Baughman, V. L., Koenig, H. M. & Albrecht, R. F. The Role of Neuronal Nitric Oxide Synthase in Regulation of Cerebral Blood Flow in Normocapnia and Hypercapnia in Rats. *Journal of Cerebral Blood Flow & Metabolism* **15**, 774–778, DOI: 10.1038/jcbfm.1995.97 (1995).

[85]Fathi, A. R. *et al.* Carbon dioxide influence on nitric oxide production in endothelial cells and astrocytes: Cellular mechanisms. *Brain Research* **1386**, 50–57, DOI: 10.1016/j.brainres.2011.02.066 (2011).

[86]Toda, N., Hatano, Y. & Mori, K. Mechanisms underlying response to hypercapnia and bicarbonate of isolated dog cerebral arteries. *American Journal of Physiology-Heart and Circulatory Physiology* **257**, H141–H146, DOI: 10.1152/ajpheart.1989.257.1.H141 (1989).

[87]Norins, N. A., Wendelberger, K., Hoffman, R. G., Keller, P. A. & Madden, J. A. Effects of Indomethacin on Myogenic Contractile Activation and Responses to Changes in O_2 and CO_2 in Isolated Feline Cerebral Arteries. *Journal of Cerebral Blood Flow & Metabolism* **12**, 866–872, DOI: 10.1038/jcbfm.1992.118 (1992).

[88]Toda, N., Ayajiki, K., Enokibori, M. & Okamura, T. Monkey cerebral arterial relaxation caused by hypercapnic acidosis and hypertonic bicarbonate. *American Journal of Physiology-Heart and Circulatory Physiology* **265**, H929–H933, DOI: 10.1152/ajpheart.1993.265.3.H929 (1993).

[89]Meigh, L. *et al.* CO2 directly modulates connexin 26 by formation of carbamate bridges between subunits. *eLife* **2013**, DOI: 10.7554/elife.01213 (2013).

[90]Huckstepp, R. T. *et al.* Connexin hemichannel-mediated CO2-dependent release of ATP in the medulla oblongata contributes to central respiratory chemosensitivity. *Journal of Physiology* **588**, 3901–3920, DOI: 10.1113/jphysiol.2010.192088 (2010).

[91]Hill, E., Dale, N. & Wall, M. J. Co2-sensitive connexin hemichannels in neurons and glia: Three different modes of signalling? *International Journal of Molecular Sciences* **22**, DOI: 10.3390/ijms22147254 (2021).

[92]Hill, E., Dale, N. & Wall, M. J. Moderate Changes in CO2 Modulate the Firing of Neurons in the VTA and Substantia Nigra. *iScience* **23**, DOI: 10.1016/j.isci.2020.101343 (2020).

[93]Altevogt, B. M. & Paul, D. L. Four Classes of Intercellular Channels between Glial Cells in the CNS. *Journal of Neuroscience* **24**, 4313–4323, DOI: 10.1523/JNEUROSCI.3303-03.2004 (2004).

[94]Xing, L. Y., Yang, T., Cui, S. S. & Chen, G. Connexin hemichannels in astrocytes: Role in CNS disorders. *Frontiers in Molecular Neuroscience* **12**, DOI: 10.3389/fnmol.2019.00023 (2019).

[95]Wells, J. A. *et al.* A Critical Role for Purinergic Signalling in the Mechanisms Underlying Generation of BOLD fMRI Responses. *The Journal of Neuroscience* **35**, 5284–5292, DOI: 10.1523/JNEUROSCI.3787-14.2015 (2015).

[96]Xu, F. *et al.* The Influence of Carbon Dioxide on Brain Activity and Metabolism in Conscious Humans. *Journal of Cerebral Blood Flow & Metabolism* **31**, 58–67, DOI: 10.1038/jcbfm.2010.153 (2011).

[97]Amadio, S., Montilli, C., Picconi, B., Calabresi, P. & Volonté, C. Mapping P2X and P2Y receptor proteins in striatum and substantia nigra: An immunohistological study. *Purinergic Signalling* **3**, 389–398, DOI: 10.1007/s11302-007-9069-8 (2007).

[98]Zhou, Z., Ikegaya, Y. & Koyama, R. The Astrocytic cAMP Pathway in Health and Disease. *International Journal of Molecular Sciences* **20**, 779, DOI: 10.3390/ijms20030779 (2019).

[99]Sinning, A. & Hübner, C. A. Minireview: pH and synaptic transmission. *FEBS Letters* **587**, 1923–1928, DOI: 10.1016/j.febslet.2013.04.045 (2013).

[100]Ruusuvuori, E. & Kaila, K. Carbonic Anhydrases and Brain pH in the Control of Neuronal Excitability. In Frost, S. C. & McKenna, R. (eds.) *Carbonic Anhydrase: Mechanism, Regulation, Links to Disease, and Industrial Applications*, vol. 75, 271–290, DOI: 10.1007/978-94-007-7359-2_14 (Springer Netherlands, Dordrecht, 2014).

[101]Lemon, N., Canepa, E., Ilies, M. A. & Fossati, S. Carbonic Anhydrases as Potential Targets Against Neurovascular Unit Dysfunction in Alzheimer's Disease and Stroke. *Frontiers in Aging Neuroscience* **13**, 772278, DOI: 10.3389/fnagi.2021.772278 (2021).

[102]Triggle, C. R. *et al.* The endothelium: Influencing vascular smooth muscle in many ways. *Canadian Journal of Physiology and Pharmacology* **90**, 713–738, DOI: 10.1139/y2012-073 (2012).

[103]Chen, B. R., Kozberg, M. G., Bouchard, M. B., Shaik, M. A. & Hillman, E. M. C. A Critical Role for the Vascular Endothelium in Functional Neurovascular Coupling in the Brain. *Journal of the American Heart Association* **3**, e000787, DOI: 10.1161/JAHA.114.000787 (2014).

[104]Zhu, J., Song, W., Li, L. & Fan, X. Endothelial nitric oxide synthase: A potential therapeutic target for cerebrovascular diseases. *Molecular Brain* **9**, 30, DOI: 10.1186/s13041-016-0211-9 (2016).

[105]Hamel, E. Perivascular nerves and the regulation of cerebrovascular tone. *Journal of Applied Physiology* **100**, 1059–1064, DOI: 10.1152/japplphysiol.00954.2005 (2006).

[106]Langer, I. Mechanisms involved in VPAC receptors activation and regulation: Lessons from pharmacological and mutagenesis studies. *Frontiers in Endocrinology* **3**, DOI: 10.3389/fendo.2012.00129 (2012).

[107]Kolluru, G. K., Shackelford, R. E., Shen, X., Dominic, P. & Kevil, C. G. Sulfide regulation of cardiovascular function in health and disease. *Nature Reviews Cardiology* **20**, 109–125, DOI: 10.1038/s41569-022-00741-6 (2023).

[108]Choi, Y. K. & Kim, Y.-M. Regulation of Endothelial and Vascular Functions by Carbon Monoxide via Crosstalk With Nitric Oxide. *Frontiers in Cardiovascular Medicine* **8**, 649630, DOI: 10.3389/fcvm.2021.649630 (2021).

[109]Ospina, J. A., Duckles, S. P. & Krause, D. N. 17β-Estradiol decreases vascular tone in cerebral arteries by shifting COX-dependent vasoconstriction to vasodilation. *American Journal of Physiology-Heart and Circulatory Physiology* **285**, H241–H250, DOI: 10.1152/ajpheart.00018.2003 (2003).

[110]Mishra, J. S., Hankins, G. D. & Kumar, S. Testosterone downregulates angiotensin II type-2 receptor via androgen receptor-mediated ERK1/2 MAP kinase pathway in rat aorta. *Journal of the Renin-Angiotensin-Aldosterone System* **17**, 147032031667487, DOI: 10.1177/1470320316674875 (2016).

[111]Tschugguel, W. *et al.* Estrogen Increases Endothelial Carbon Monoxide, Heme Oxygenase 2, and Carbon Monoxide-Derived cGMP by a Receptor-Mediated System. *The Journal of Clinical Endocrinology & Metabolism* **86**, 3833–3839, DOI: 10.1210/jcem.86.8.7715 (2001).

[112]Lopes, R. A. M., Neves, K. B., Carneiro, F. S. & Tostes, R. C. Testosterone and Vascular Function in Aging. *Frontiers in Physiology* **3**, DOI: 10.3389/fphys.2012.00089 (2012).

[113]Akishita, M. & Yu, J. Hormonal effects on blood vessels. *Hypertension Research* **35**, 363–369, DOI: 10.1038/hr.2012.4 (2012).

[114]Deer, R. R. & Stallone, J. N. Effects of estrogen on cerebrovascular function: Age-dependent shifts from beneficial to detrimental in small cerebral arteries of the rat. *American Journal of Physiology-Heart and Circulatory Physiology* **310**, H1285–H1294, DOI: 10.1152/ajpheart.00645.2015 (2016).

[115]Mendelsohn, M. E. & Rosano, G. M. Hormonal Regulation of Normal Vascular Tone in Males. *Circulation Research* **93**, 1142–1145, DOI: 10.1161/01.RES.0000108694.68635.1C (2003).

APPENDIX: TABLE OF ACRONYMS

**$\alpha_{\#}$Ar**	α-# Adrenergic receptor	**βAr**	β-Adrenergic receptor
**AA**	Arachidonic Acid	**ACh**	Acetylcholine
**ACyc**	Adenylyl Cyclase	**Adeno**	Adenosine
**AMPAr**	Alpha-amino-3-hydroxy-5-Methyl-4-isoxazolepropionic Acid receptor	**ASIC**	Acid Sensing Ion Channel
**AT**	Angiotensin	**AT$_{\#}$r**	Angiotensin $_{\#}$ receptor
**ATP**	Adenosine Triphosphate	**BK**	Big Potassium
**BrK**	Bradykinin	**Ca**	Calcium (atomic symbol)
**CA**	Carbonic Anhydrase	**CaM**	Calmodulin
**cAMP**	Cyclic Adenosine Monophosphate	**cGMP**	Cyclic Guanine Monophosphate
**CGRP**	Calcitonin Gene Related Peptide	**CICR**	Calcium Induced Calcium Release
**CMRO$_2$**	Cerebral Metabolic Rate of Oxygen Consumption	**COX(1/2)**	Cyclooxygenase (1/2)
**CO**	Carbon Monoxide	**CO$_2$**	Carbon Dioxide
**CP450(E/H)**	Cytochrome P450 (Epoxygenase/Hydroxylase)	**CxH**	Connexin Hemichannel
**CSF**	Cerebral Spinal Fluid	**DAG**	Diacylglycerol
**E**	Estrogen	**EET**	Epoxyeicosatrienoic acid
**ER**	Endoplasmic Reticulum	**EP4**	E-type Prostanoid receptor 4
**eNOS**	Endothelial Nitric Oxide Synthase	**GABA**	Gamma-Aminobutyric Acid
**GABA$_{A/B}$**	Gamma-Aminobutyric Acid A/B Receptor	**Glu**	Glutamate
**GPCR**	G-protein-coupled Receptor	**H$_2$S**	Hydrogen Sulfide
**HRF**	Hemodynamic Response Function	**HO**	Heme-oxygenase
**iGluR**	Ionotropic Glutamate Receptor	**IP3(r)**	Inositol Triphosphate (Receptor)
**K**	Potassium (Atomic Symbol)	**K$_{ir}$**	Inward Rectifying Potassium Channel
**NA**	Noradrenaline	**Na**	Sodium (Atomic Symbol)
**NBCe1**	Electrogenic Sodium Bicarbonate Cotransporter 1	**nAChr**	Nicotinic Acetylcholine receptor
**NMDAr**	N-Methyl-D-Aspartate receptor	**NO**	Nitric Oxide
**NT**	Neurotransmitter	**nNOS**	Neuronal Nitric Oxide Synthase
**mAr**	Metabotropic Adenosine Receptor	**mGlur**	Metabotropic Glutamate Receptor
**mAChr**	Muscarinic Acetylcholine receptor	**MLCK**	Myosin Light Chain Kinase
**MLCP**	Myosin Light Chain Phosphatase	**NPY(r)**	Neuropeptide Y (receptor)
**NVC**	Neurovascular Coupling	**NVU**	Neurovascular Unit
**O$_2$**	Oxygen	**PDE#**	Phosphodiesterase-#
**PGE2**	Prostaglandin E2	**PI3K**	Phosphotidylinositol-3-Kinase
**PIP2**	Phosphotidylinositol 4,5-Bisphosphate	**PK(A/C/G)**	Protein Kinase A/C/G
**PL(A2/C/D2)**	Phospho-lipase (A2/C/D2)	**PGS**	Prostaglandin Synthase
**PVS**	Perivascular Space	**Ryr**	Ryanodine Receptor

**sGC**	**S**oluble **G**uanyl **C**yclase	**SACC**	**S**tretch **A**ctivated **C**ation **C**hannel
**SR**	**S**arcoplasmic **R**eticulum	**T**	**T**estosterone
**TP**	**T**hromboxane receptor	**TX**	**T**hrombo**x**ane
**TXS**	**T**hrombo**x**ane **S**ynthase	**VIP**	**V**asoactive **I**ntestinal **P**eptide
**VOCC**	**V**oltage **O**perated **C**alcium **C**hannel	**VSM**	**V**ascular **S**mooth **M**uscle
**WSS**	**W**all **S**hear **S**tress		

Table S3: Acronyms used in the interaction graphs and throughout the manuscript and supplementary material.
